# Decoration of 1,4,7,10‐tetraazacyclododecane‐1,4,7,10‐tetraacetic acid (DOTA) with *N*‐oxides increases the *T*_1_ relaxivity of Gd‐complexes

**DOI:** 10.1002/open.202300298

**Published:** 2024-01-15

**Authors:** Svenja Kerpa, Verena R. Schulze, Malte Holzapfel, Lina Cvancar, Markus Fischer, Wolfgang Maison

**Affiliations:** ^1^ Department of Chemistry Institute of Pharmacy Universität Hamburg Bundesstrasse 45 20146 Hamburg Germany; ^2^ Fraunhofer Institute for Applied Polymer Research IAP Center for Applied Nanotechnology CAN Universität Hamburg Bundesstrasse 45 20146 Hamburg Germany; ^3^ Hamburg School of Food Science Institute of Food Chemistry University of Hamburg Grindelallee 117 20146 Hamburg Germany

**Keywords:** Gadolinium based contrast agents, *T*_1_ relaxivity, *N*-oxides, magnetic resonance imaging (MRI), Tetraazacyclododecane-1,4,7,10-tetraacetic acid (DOTA), Cu-catalyzed azide alkyne cycloaddition (CuAAC)

## Abstract

High complex stability and longitudinal relaxivity of Gd‐based contrast agents are important requirements for magnetic resonance imaging (MRI) because they ensure patient safety and contribute to measurement sensitivity. Charged and zwitterionic Gd^3+^‐complexes of the well‐known chelator 1,4,7,10‐tetraazacyclododecane‐1,4,7,10‐tetraacetic acid (DOTA) provide an excellent basis for the development of safe and sensitive contrast agents. In this report, we describe the synthesis of DOTA‐NOx, a DOTA derivative with four *N*‐oxide functionalities *via* “click” functionalization of the tetraazide DOTAZA. The resulting complexes Gd‐DOTA‐NOx and Eu‐DOTA‐NOx are stable compounds in aqueous solution. NMR‐spectroscopic characterization revealed a high excess of the twisted square antiprismatic (TSAP) coordination geometry over square antiprismatic (SAP). The longitudinal relaxivity of Gd‐DOTA‐NOx was found to be *r*_1_=7.7 mm ^−1^ s^−1^ (1.41 T, 37 °C), an unusually high value for DOTA complexes of comparable weight. We attribute this high relaxivity to the steric influence and an ordering effect on outer sphere water molecules surrounding the complex generated by the strongly hydrated *N*‐oxide groups. Moreover, Gd‐DOTA‐NOx was found to be stable against transchelation with high excess of EDTA (200 eq) over a period of 36 h, and it has a similar *in vitro* cell toxicity as clinically used DOTA‐based GBCAs.

## Graphical abstract

**Decoration with** ***N*** **‐oxide groups** is a valuable method to increase the *T*_1_‐relaxivity of the well‐known cyclic Gd‐chelator DOTA. The synthesis of Gd‐DOTA‐NOx has been achieved *via* copper catalyzed alkyne azide cycloaddition. The complex is water soluble, stable against transchelation, has a twisted square antiprismatic (TSAP) complex geometry and a high *T*_1_‐relaxivity of 7.7 mm ^−1^ s^−1^ (1.41 T, 37 °C).

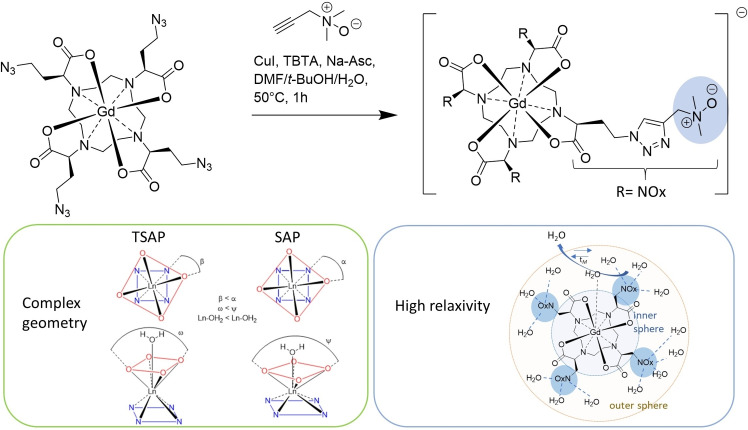

## Introduction

**Scheme 1:**
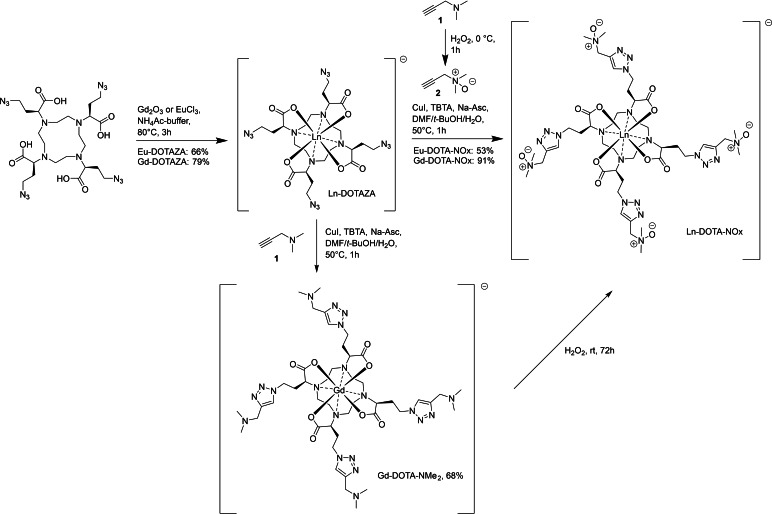
Two different approaches to Ln‐DOTA‐NOx starting from Ln‐DOTAZA *via* CuAAC with propargyl‐*N*‐oxide **2** or propargylamine **1**.

Magnetic resonance imaging (MRI) is one of the most important methods in diagnostic imaging. Despite the widespread clinical use of MRI, the method has a limited sensitivity and can thus not easily be adapted to targeted applications.^[1]^ In *T*_1_ weighted imaging, paramagnetic contrast agents shorten *T*_1_ (also called the spin‐lattice relaxation time) of tissue in which they accumulate. These regions have therefore a brighter signal. Gadolinium‐based contrast agents (GBCAs) are primarily used in this context although other metals are also attractive.^[2]^ However, gadolinium is a toxic heavy metal and potentially leads to nephrogenic systemic fibrosis in patients with impaired renal clearance. In addition, it has occasionally been found to accumulate in the brain, although the physiological consequences of the latter process are not completely clear at this stage.^[3]^ Therefore, regulatory agencies (e. g. the FDA and EMA) recommended to suspend the use of GBCAs with linear chelators (such as diethylenetriaminepentaacetic acid (DTPA)) or restrict their use to limited applications. Cyclic chelators, in contrast, lead typically to more stable Gd^3+^‐complexes and are thus preferred in clinical applications. Overall, stability is therefore a key issue for the design of chelators for GBCAs to avoid the release of toxic Gd^3+^ *in vivo*.^[2a]^ Cyclic chelators like 1,4,7,10‐tetraazacyclododecane‐1,4,7,10‐tetraacetic acid (DOTA, scheme 1) and derivatives thereof form highly stable Gd‐complexes.^[4]^ They are therefore most frequently used for MRI.^[5],^ ^[6]^ Next to various other important parameters, such as solubility and tissue distribution, the relaxivity of these complexes is an important parameter to consider.^[7]^ The development of new GBCAs with high relaxivity is therefore an attractive research area. Relaxivity describes the extent to which a GBCA changes the relaxation rate of ^1^H nuclei of surrounding water molecules.^[8]^ For a given GBCA it depends on external factors such as temperature and applied field. In addition, it is dependent on the number and exchange rate of water molecules in the inner and the outer sphere of Gd^3+^‐complexes. The number of inner sphere water molecules (hydration number q) is specific for each complex and cannot easily be changed for a given chelator. For Gd‐DOTA derivatives q is typically 1.^[9]^

**Figure 1:**
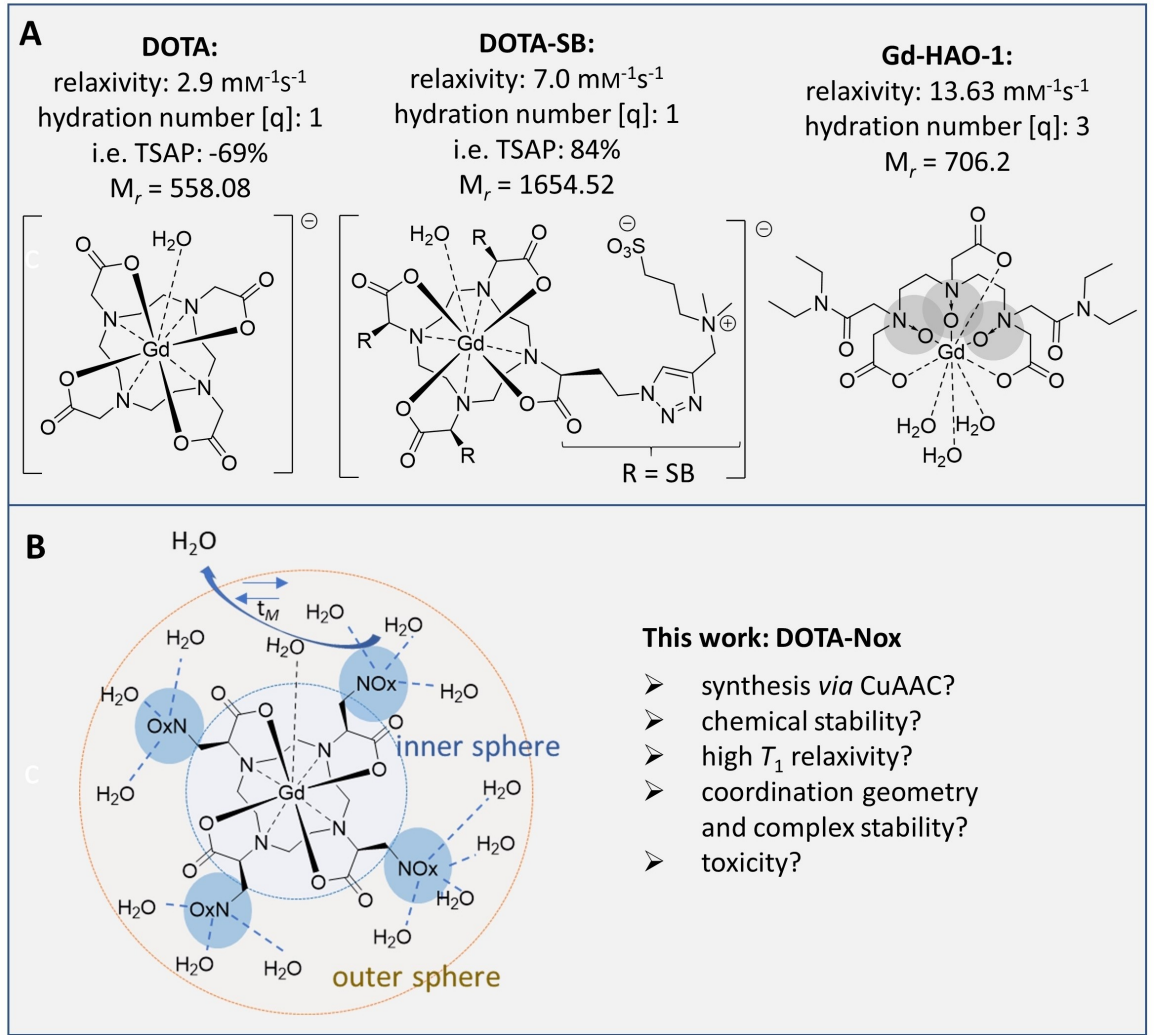
A: Chemical structure, relaxivity (*r_1_*), hydration number (q), excess of TSAP‐isomer (% i. e.) and molecular weight of three known Gd‐complexes: Gd‐DOTA,^[14]^ Gd‐DOTA‐SB and Gd‐HAO‐1.^[15]^ The isomeric excess of TSAP (i. e. in %) is given for the corresponding Eu‐complexes. B: Schematic drawing of Gd‐DOTA‐NOx with associated water in the inner and outer sphere.

The *T*_1_ relaxivity of GBCAs of low molecular weight is often limited by their rotational correlation time. However, if complex rotation is slowed down (through an increase in Stokes radius), water exchange may become a limiting factor. This process is particularly important at low field strength <3 T and is less important for high fields >3 T.^[10]^ The inner sphere water exchange depends strongly on the complex geometry.[^7^, ^11^] Gd^3+^‐DOTA complexes can exist as two different stereoisomers: a compact square antiprismatic (SAP) and an elongated twisted square antiprismatic (TSAP) isomer. Sterically demanding substituents in the sidechains of DOTA cause a longitudinal distortion of the coordination polyeder and favor the TSAP isomer.^[12]^ This leads to a relatively long Gd‐OH_2_ distance and an increase in the inner sphere water exchange rate and thus high relaxivity of the complex. This way, an increase in TSAP isomer over SAP isomer correlates typically to an increase in relaxivity. As mentioned above, the rotational correlation time is also an important parameter for the relaxivity. It is influenced by the order of outer sphere water molecules which can be altered by substituents attached to the DOTA core structure. Charged residues and particularly zwitterionic groups have been found to have the largest impact on the relaxivity of Gd‐DOTA complexes of low molecular weight.[^6a^, ^13^] Figure 1 highlights the influence of zwitterionic decoration and the hydration number of Gd‐complexes on *T*_1_ relaxivity. GBCAs in clinical use have typically *T*_1_ relaxivities of 2–4 mm ^−1^ s^−1^ (e. g. [Gd‐DOTA]^1−^ (gadoteric acid, DOTAREM®) *r*_1_=2.9 mm ^−1^ s^−1^ (1.41 T, 37 °C) or [Gd‐DTPA]^2−^ (gadopentetic acid, Magnevist®) *r*_1_=3.4 mm ^−1^ s^−1^ (1.41 T, 37 °C)). An increase in the hydration number of Gd‐complexes leads to a substantial increase in *T*_1_ relaxivity. A good example is Gd‐HAO‐1 (Figure 1) which has an increased hydration number and an increased *T*_1_ relaxivity compared to the parent compound Gd‐DTPA. However, an increase in the hydration number comes typically with a decreased complex stability, which is reflected by the absence of clinically used GBCAs with hydration numbers significantly higher than 1. On the other hand, *T*_1_ relaxivity can be enhanced by the introduction of zwitterionic groups without influencing the hydration number (and thus the complex stability). A recent example is Gd‐DOTA‐SB (SB=sulfobetaine), which has a significantly higher *T*_1_ relaxivity than Gd‐DOTA. It should be noted that a number of other factors affect the *T*_1_ relaxivity of GBCAs. It is therefore not easy to separate their contribution. The increased molecular mass of Gd‐DOTA‐SB, for example, has been demonstrated to have only a minor impact on the relaxivity of this compound.^[6a]^

*N*‐oxides are special zwitterionic structures easily prepared by oxidation of tertiary amines.^[16]^ With their dative N^+^−O^−^ bond, they have the shortest possible spacer between the two separate charges. Consequently, they have large dipole moments of 4–5 D^[17]^ and can form strong hydrogen bonds.^[18]^ This makes *N*‐oxides powerful kosmotropes, a property used increasingly in various healthcare and technical applications.^[19]^ Different *N*‐oxides are also found in nature. Trimethylamine oxide (TMAO), for example, is a natural *N*‐oxide found in seawater fish (and also in humans^[20]^).^[21]^ TMAO stabilizes proteins by counteracting protein‐denaturing compounds (eg. chaotropes like urea) or denaturing forces like heat and pressure. It is also notable that *N*‐oxides of alkaloids are non‐toxic derivatives of their toxic membrane‐permeable parent amines and are used for vesicle storage in plants and insects.^[22]^ Moreover, polymeric *N*‐oxides have been shown to be blood compatible and have low fouling properties.^[23]^ The introduction of *N*‐oxide groups is thus a promising method to develop Gd‐DOTA complexes with high relaxivity, good water solubility and low toxicity.

However, several *N*‐oxides have a high chemical reactivity.^[18b]^ This is reflected by their common use as oxidants and intermediates in organic synthesis[^18b^, ^24^] as well as biosynthesis.^[25]^ Several *N*‐oxides are biologically active and have been explored as drugs.[^22c^, ^26^] Reductive metabolism by microorganisms or in hypoxic tissue triggers their biological activity as antibiotics or cytotoxic compounds for chemotherapy.^[27]^ In addition, *N*‐oxides are good complex ligands for several transition metals.^[28]^ This has been exploited with the synthesis of *N*‐oxide derivatives of DOTA^[29]^ and other chelators, such as DTPA (e. g. HAO‐1, Figure 1).[^15^, ^30^] In these cases, the *N*‐oxides are involved in coordinating the central metal ion. The resulting metal complexes like Gd‐HAO‐1 do thus not contain “free” *N*‐oxide groups but rather coordinated *N*‐oxides. Although Gd‐complexation by *N*‐oxide side chains of DOTA‐NOx presented in this work cannot be excluded *a priori*, it was designed to keep the standard metal binding motif of DOTA. The “decorating” *N*‐oxides in the pendant arms of DOTA are thus unlikely involved in metal coordination and are therefore available for strong water binding. The latter aspect might improve the relaxivity of the complex due to an increase in hydrodynamic diameter and an influence on the exchange of inner and outer sphere water molecules.

The most important questions addressed by this study are thus: 1. How can DOTA be decorated with *N*‐oxide functionalities retaining the central amino acid binding motif for Gd^3+^? 2. Is the resulting DOTA‐NOx chemically stable? 3. Do the *N*‐oxide groups have an impact on the geometry and relaxivity of the corresponding Gd^3+^‐complexes? And 4. Is Gd‐DOTA‐NOx toxic to human cell lines?

## Results and Discussion

### Synthesis of DOTA‐NOx

The tetraazido derivative DOTAZA (Scheme 1) has been used previously for the synthesis of various sidechain substituted DOTA derivatives.[^6a^, ^12b^, ^31^] It allows the introduction of *N*‐oxide groups by two different routes which include either the conjugation of a preformed *N*‐oxide derivative *via* Cu‐catalyzed azide alkyne cycloaddition (CuAAC) or the conjugation of a tertiary amine and the subsequent oxidation to *N*‐oxides. Both routes have possible drawbacks because 1) the oxidative reactivity of *N*‐oxides might be incompatible with CuAAC involving Cu(I)‐catalysis and 2) the tertiary amines of the cyclen scaffold might lead to regioselectivity problems if the *N*‐oxides are generated by a final oxidation. The oxidation of the cyclen nitrogens in DOTA is to the best of our knowledge not known. However, similar oxidations have been performed with acyclic chelators like DTPA by Hu and coworker.[^15^, ^30^]

In a first approach, DOTAZA was treated with Gd_2_O_3_ or EuCl_3_ to give the corresponding complexes Gd‐DOTAZA and Eu‐DOTAZA in 79 % and 66 % yield respectively (Scheme 1).^[12b]^ The Eu‐complex was prepared for NMR‐spectroscopic evaluation of the coordination geometry. The following CuAAC was performed under standard conditions and gave the *N*‐oxides Gd‐DOTA‐NOx in 91 % and Eu‐DOTA‐NOx in 53 % yield after chromatographic purification. The propargyl‐*N*‐oxide **2** was prepared by oxidation of *N,N*‐dimethylpropargylamine **1** with H_2_O_2_ following a literature procedure.^[32]^ It should be noted that **2** is not stable for longer periods of time at room temperature and is best stored at 0–5 °C in aqueous solution. It is also essential that all remaining H_2_O_2_ is quenched with MnO_2_ after the reaction to avoid Cu(I)‐oxidation and thus catalyst deactivation in the following CuAAC. The CuAAC of Ln‐DOTAZA to Ln‐DOTA‐NOx proceeded in high yield. However, the reaction conditions needed to be controlled carefully. It was particularly important to keep the reaction temperature at 50 °C and the reaction time below 1 h. Longer reaction times or higher temperatures lead to an increase in side reactions. This reflects the redox properties of *N*‐oxides, which are known to be reduced to tertiary amines by Cu(I).^[33]^ The reduction of Ln‐DOTA‐NOx was found to be significantly slower than its formation *via* CuAAC. However, if the reaction time was extended to 4 h, significant quantities of the corresponding tertiary amines derived from reduction of Gd‐DOTA‐NOx were observed by LC/MS analysis.

An alternative approach *via* CuAAC of Gd‐DOTAZA with propargylamine **1** to Gd‐DOTA‐NMe_2_ and subsequent oxidation to Gd‐DOTA‐NOx with H_2_O_2_ was also explored (Scheme 1). This approach avoids the preparation of the relatively unstable intermediate *N*‐oxide **2**. Again, the CuAAC to the intermediate amine Gd‐DOTA‐NMe_2_ worked fine, but the final oxidation to Gd‐DOTA‐NOx turned out to be extremely slow. Mixtures of Gd‐DOTA‐NOx and incompletely oxidized derivatives with 1–3 *N*‐oxide groups in the pendant arms were observed by LC/MS analysis even after a long reaction time of 72 h. The separation of these mixtures by chromatography was impossible due to the high polarity of the compounds and coresponding poor retention on RP columns making this route inconvenient for the preparation of DOTA‐NOx complexes.

The CuAAC of Gd‐DOTAZA with propargyl‐*N*‐oxide **2** was thus the most efficient route to Gd‐DOTA‐NOx. It is notable that the CuAAC is compatible with *N*‐oxide moieties. However, the reaction conditions need to be carefully controlled to avoid competing reductions of *N*‐oxide groups by Cu(I). The target compounds Ln‐DOTA‐NOx were found to be rather chemically stable and can be stored for weeks at 0–5 °C in pure form or in aqueous solution without decomposition.

### Complex geometry of Eu‐DOTA‐NOx

As mentioned above, the coordination geometry of Gd‐complexes has a strong impact on their relaxivity.^[34]^ The TSAP/SAP ratio in Ln‐DOTA complexes refers to two coordination isomers: a square antiprismatic geometry (SAP) and a twisted square antiprismatic geometry (TSAP).^[35]^ An increase in the size of substituents in the pendant arms of DOTA leads typically to a longitudinal distortion of the coordination polyeder and thus an increase in the amount of TSAP isomer of the complex. The TSAP/SAP ratio of DOTA complexes can be measured by ^1^H‐NMR of Eu‐complexes, which are known to be good NMR‐accessible model compounds of the corresponding Gd‐complexes.^[36]^ The large lanthanide shift of the axial protons in the cyclen ring leads to a significant chemical shift difference for these protons resulting in a downfield signal for the SAP (30‐45 ppm) and an upfield signal for the TSAP (10‐25 ppm) protons. Both isomers are thus easily distinguishable by their characteristic shifts and may be quantified by integration of the signals. The ratio of the integrals for the two isomers TSAP and SAP is given as percentage isomeric excess of the TSAP isomer (% i. e.). Values can therefore also assume negative values (for TSAP<SAP).

**Figure 2:**
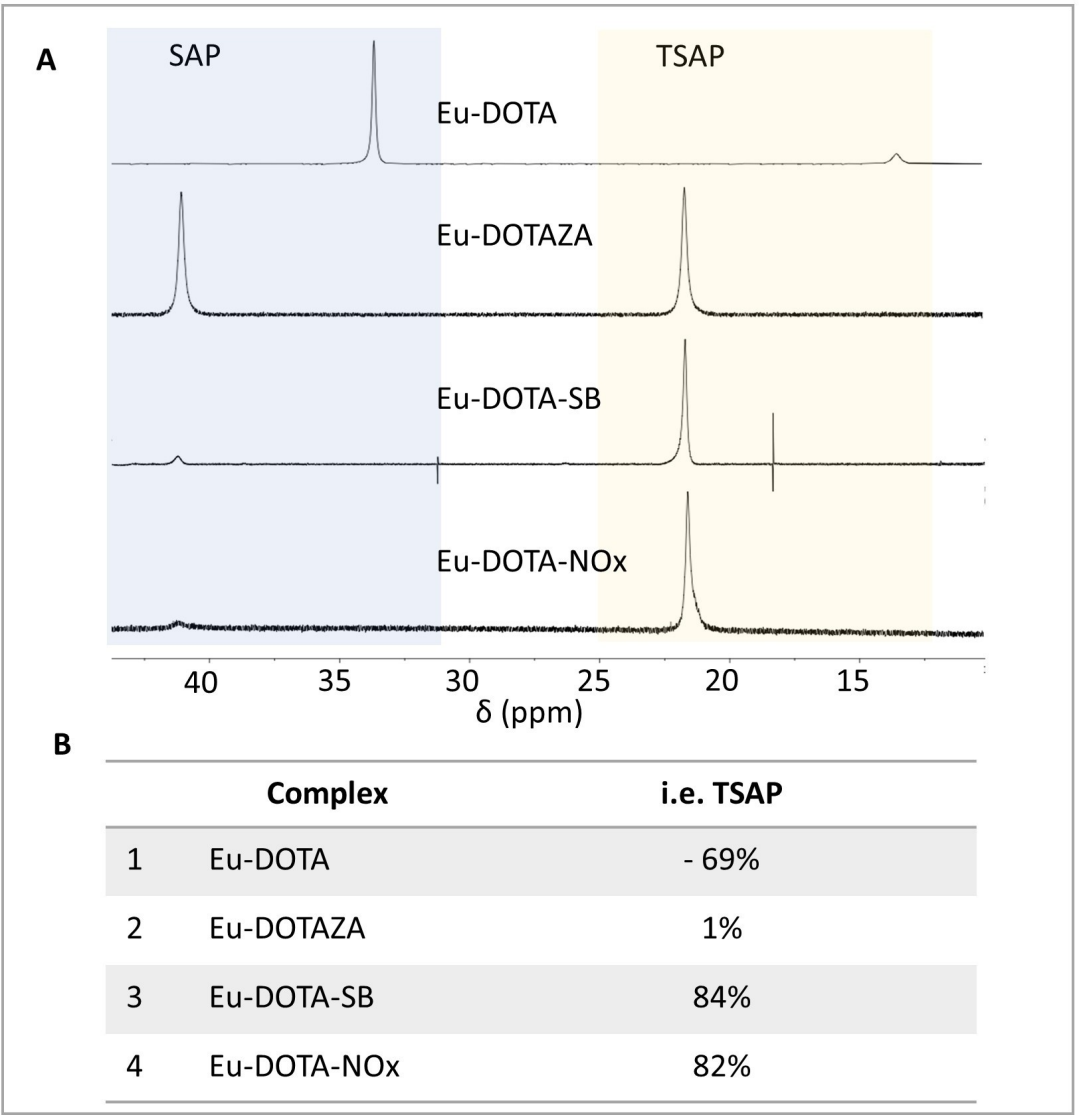
A: Stretches of ^1^H‐NMR spectra between δ=45‐10 ppm for Eu‐DOTA, Eu‐DOTAZA, EU‐DOTA‐SB, Eu‐DOTA‐NOx in D_2_O at 298 K and 400 MHz. B: Isomeric excess of TSAP‐isomer (% i. e.) as measured by integration of ^1^H‐NMR spectra.

To this end, Eu‐DOTA‐NOx was analyzed by ^1^H NMR revealing a significant excess of TSAP isomer (82 %, Figure 2). This preference for the TSAP isomer is similar to that obtained for a previously described Eu‐DOTA‐SB bearing sulfobetaine groups in the pendant arms (Eu‐DOTA‐SB: 84 % i. e. TSAP) and is significantly higher compared to Eu‐DOTAZA (1 % i. e. TSAP) and Eu‐DOTA (−69 % i. e. TSAP).^[6a]^ The high preference of Eu‐DOTA‐SB and Eu‐DOTA‐NOx for the TSAP coordination geometry is most likely reflecting the bulkiness of the zwitterionic side chains in these derivatives. The NMR spectra of Eu‐DOTA‐NOx are quite similar to those of other Eu‐DOTA derivatives with substituents in the four pendent arms (spectra of Eu‐DOTAZA and Eu‐DOTA‐SB are depicted in Figure 2A for comparison). This supports the proposed coordination of lanthanides by the amino acid core structure of DOTA‐NOx without coordination of the *N*‐oxides. Unfortunately, we have not been able to confirm this hypothesis with a X‐ray crystal structure analysis. In our hands, Ln‐DOTA‐NOx complexes did not form crystals, which might be due to their conformational flexibility and hygroscopic properties.

### Relaxivity of Gd‐DOTA‐NOx

The relaxivity of Gd‐DOTA‐NOx was analyzed at a magnetic field strength of 1.41 T at 37 °C. Briefly, the longitudinal relaxation time *T*_1_ was determined in an inversion‐recovery experiment. *T*_1_ was measured for a dilution series (0.65, 3.25 and 6.50 mm) of Gd‐DOTA‐NOx in H_2_O. The longitudinal relaxivity *r_1_* was derived from a reciprocal plot of *T*_1_ against the concentration *c* by linear regression. A precise determination of the Gd‐concentration is therefore crucial to get reliable results. This is particularly challenging for highly hygroscopic compounds like Gd‐DOTA‐NOx, that contained varying amounts of water. The Gd‐concentrations of the solutions were therefore precisely confirmed by inductively coupled plasma (ICP) mass spectrometric (MS) analysis.

**Figure 3:**
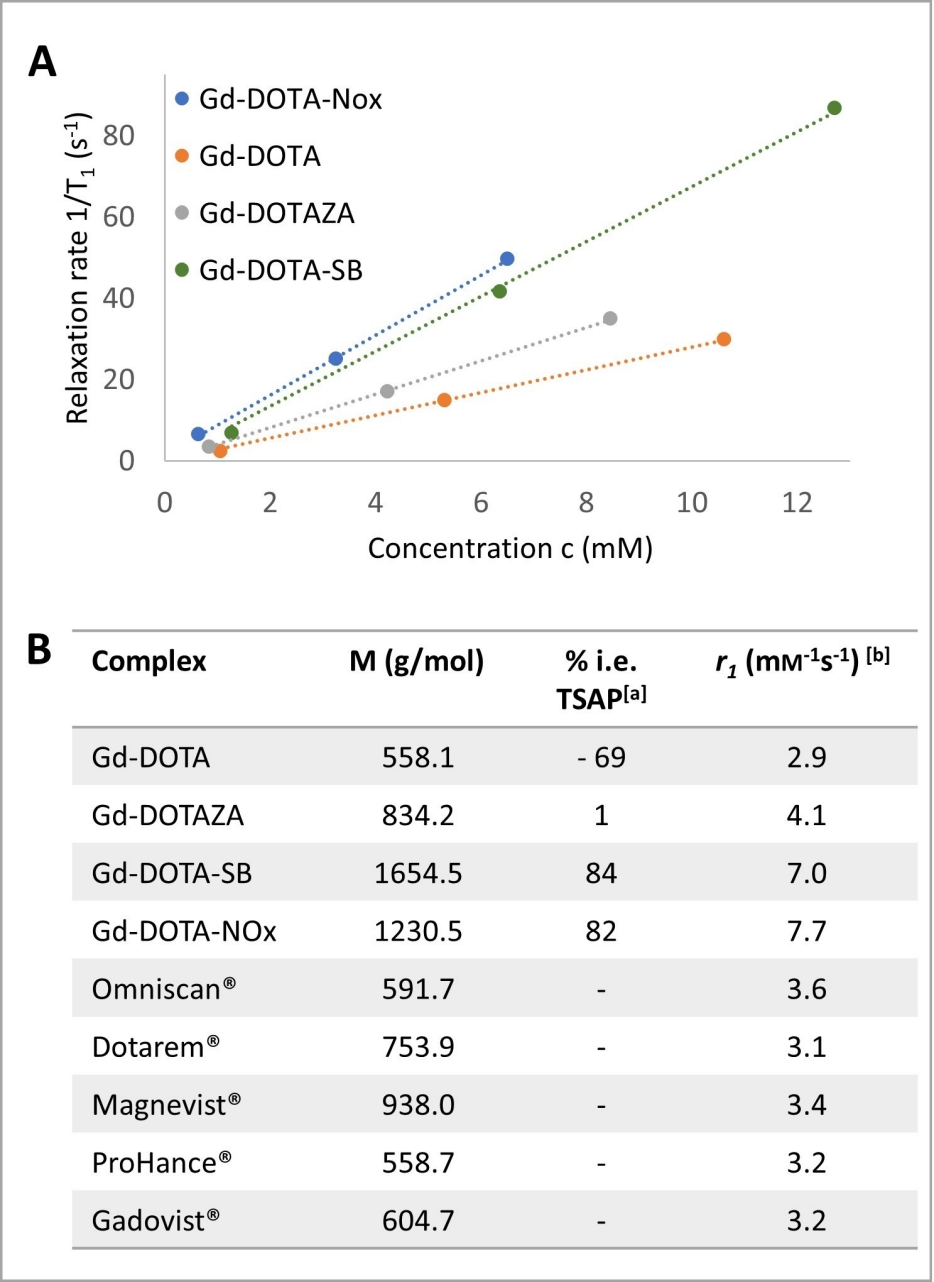
**A**: Plot of relaxation rate against the concentration (mM) of Gd‐DOTA‐NOx, Gd‐DOTA, Gd‐DOTAZA, Gd‐DOTA‐SB. Measurements were performed at 1.41 T, 37 °C in H_2_O. **B**: Molecular weight (g/mol), isomeric excess of TSAP (% i. e. TSAP) and relaxivity (mM^−1^s^−1^) for selected DOTA complexes. ^[a]^ Isomeric excess of TSAP isomer for Eu‐complexes as measured by integration of the ^1^H‐NMR spectra. ^[b]^ Measured at 1.41 T, 37 °C in H_2_O.

The resulting longitudinal relaxivities are depicted in Figure 3 for Gd‐DOTA‐NOx and for comparison Gd‐DOTA and Gd‐DOTA‐SB.^[6a]^ Gd‐DOTA has the lowest value in this series with *r*_1_=2.9 mm ^−1^ s^1^, which is a typical value for DOTA‐complexes in clinical use.^[37]^ These complexes are of low molecular weight and are either neutral or have only one negative charge. In addition, they prefer the SAP coordination geometry. In contrast, Gd‐DOTA‐SB was found to have an exceptionally high relaxivity of *r_1_* =7.0 mm ^−1^ s^−1^ (1.41 T, 37 °C). As mentioned above, the relaxivity of structurally related complexes depends on the molecular weight of the complex, its hydrodynamic diameter and the coordination geometry. We have noted previously, that the high relaxivity of Gd‐DOTA‐SB can be explained to a limited extend by its preference for the TSAP coordination (i. e. TSAP=84 % as measured for the corresponding Eu‐complex). The increase in molecular mass compared to Gd‐DOTA cannot account for the large increase in relaxivity. The most important factor for the high relaxivity of Gd‐DOTA‐SB is most likely the increase in hydrodynamic diameter through the four sulfobetaine residues, which increase the Stokes radius of the molecule. This reflects the strong hydration which is typical for sulfobetaine zwitterions. An even higher relaxivity of *r*_1_=7.7 mm ^−1^ s^−1^ (1.41 T, 37 °C) was observed for Gd‐DOTA‐NOx, which is the highest value we have determined for DOTA‐complexes of low molecular weight so far. Gd‐DOTA‐NOx has a similar preference for TSAP coordination (i. e. TSAP=82 % as measured for the corresponding Eu‐complex) and a lower molecular mass than Gd‐DOTA‐SB. The increased relaxivity of Gd‐DOTA‐NOx can thus most likely be attributed to the strong hydration of the four *N*‐oxide groups attached to the DOTA pendant arms. *N*‐oxides are known kosmotropes and are typically surrounded by a well‐ordered layer of solvent water molecules. This property of *N*‐oxides has not only a positive influence on the relaxivity of Gd‐complexes. It is also responsible for the stealth character of poly‐*N*‐oxides which has recently been exploited in medicinal chemistry to improve blood compatibility and low fouling properties of drugs and materials.^[23b]^

### Kinetic stability and cytotoxicity

As mentioned before, a key factor for the development of new GBCAs is complex stability. A lack of stability may result in release of Gd^3+^ *in vivo* which can lead to nephrogenic systemic fibrosis or gadolinium deposition disease.[^3^, ^38^] *In vivo*, kinetic instability of Gd‐complexes is a result of metal transchelation.^[39]^ This process is hard to imitate with *in vitro* experiments. However, the stability of metal complexes for imaging is commonly assessed by transchelation assays with high excess of competing chelators such as EDTA.^[40]^

**Figure 4:**
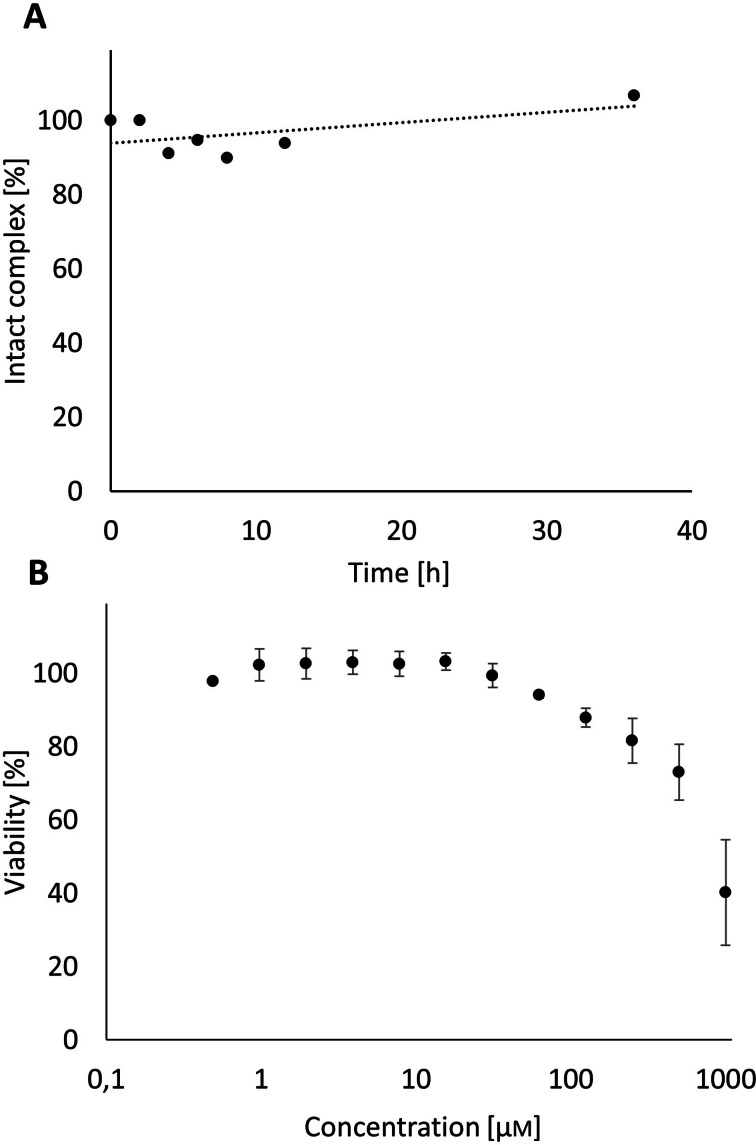
A: Transchelation assay of Gd‐DOTA‐NOx challenged with a 200‐fold excess of EDTA at pH 7.4 at room temperature. The fate of the intact Gd‐complex was followed *via* LC/ESI‐MS over 36 h. A sample of pure Gd‐DOTA‐NOx was measured as an external standard at 0 h and 36 h. B: Cell viability [%] of HeLa after treatment with Gd‐DOTA‐NOx at different concentrations between 0.5‐1000 μm. Data are means±standard deviation for three independent experiments.

All of the data obtained so far pointed towards a standard complex geometry in Gd‐DOTA‐NOx similar to other thermodynamically and kinetically stable Gd‐DOTA‐complexes. A compromised complex stability of Gd‐DOTA‐NOx was therefore not anticipated. However, the stability of Gd‐DOTA‐NOx was evaluated anyhow using a competition assay with EDTA and LC/MS monitoring. Briefly, the Gd‐complex was treated with a 200‐fold excess EDTA in HEPES buffer (pH 7.4). The fate of the intact Gd‐complex was followed *via* LC/MS over 36 h.^[40]^ The resulting data in Figure 4 reveals a high kinetic stability of Gd‐DOTA‐NOx for at least 36 h at room temperature. This is in line with the expectations for a DOTA derivative and is additional evidence for the coordination of Gd^3+^ by the standard amino acid coordination motif of DOTA‐NOx.^[5b]^ The complex stability of Gd‐DOTA‐NOx is thus not significantly compromised by the decoration of DOTA with *N*‐oxides.

The cytotoxicity of Gd‐DOTA‐NOx was also evaluated. For this purpose, the cell viability of HeLa cells was measured after treatment with Gd‐DOTA‐NOx at concentrations of 0.5‐1000 μm over a period of 24 h in a 96‐well plate at 37 °C and 5 % CO_2_ with complete DMEM media. Resazurin in cell media was added and after 3 h the amount of resorufin measured colorimetrically (560/590 nm).

The resulting data is depicted in Figure 4B. It reveals a loss of cell viability at Gd‐DOTA‐NOx concentrations higher than 100 μm. Considering the fact, that the local concentration of GBCAs after administration is often higher than 100 μm to achieve good contrast in target tissue, the observed cytotoxicity seems to compromise future *in vivo* applications. However, similar cytotoxicities have been found for a range of clinically used DOTA‐based GBCAs *in vitro*.^[41]^ The observed cytotoxicity of Gd‐DOTA‐NOx is therefore not necessarily related to the *N*‐oxide functionalities but might be an intrinsic property of many Gd‐DOTA complexes which does not compromise *in vivo* applications. However, given the special reactivity profile of *N*‐oxides in chemical reactions and also in several *in vivo* applications (see introduction) this point might deserve more detailed future studies.^[23a]^

## Conclusions

This paper introduces the decoration of the cyclic chelator DOTA with *N*‐oxide functionalities. The latter are known to be strongly hydrated zwitterionic groups in aqueous solution and might thus have favorable properties for the development of new GBCAS. The synthesis of Gd‐DOTA‐NOx has been accomplished by CuAAC of the known precursor DOTAZA. Particular attention should be paid to the removal of H_2_O_2_ traces from the propargyl‐*N*‐oxide used as the alkyne component. In addition, the reaction temperature needs to be kept at 50 °C and the reaction time below 1 h to avoid the competing reduction of *N*‐oxides with Cu(I). Eu‐DOTA‐NOx was analyzed as a model compound *via* NMR spectroscopy. These studies revealed a high preference for the TSAP isomer (82 % i. e. TSAP) similar to a recently reported sulfobetaine derivative of DOTA. This supports the proposed coordination of lanthanides by the amino acid core structure of DOTA‐NOx without coordination of the *N*‐oxides. The *T*_1_ relaxivity of Gd‐DOTA‐NOx was found to be *r*_1_=7.7 mm ^−1^ s^−1^ at 1.4 T and 37 °C, the highest value we have observed for DOTA derivatives of comparable molecular weight. The increased relaxivity of Gd‐DOTA‐NOx might be attributed to the strong hydration of the four *N*‐oxide groups attached to the DOTA pendant arms. Gd‐DOTA‐NOx was found to be kinetically stable against competition with 200‐fold excess EDTA by an HPLC/MS assay. *In vitro* viability tests with HeLa‐cells revealed similar cytotoxicity as other clinically used DOTA‐based GBCAs.

In view of these results, the “decoration” of metal chelators (DOTA and others) with *N*‐oxide functionalities might be an attractive method to improve the relaxivities of GBCAs. Given the special physical and chemical properties of *N*‐oxides, the resulting imaging reagents might have added advantageous properties such as good solubility, stealth character and probably also redox activity *in vivo*. However, the special reactivity profile of *N*‐oxides in chemical reactions and also in several *in vivo* applications might deserve more detailed future studies on their *in vitro* and *in vivo* toxicity.

## Experimental Section

**General**. Reagents and starting materials were purchased by commercial sources and used without further purification. All non‐deuterated organic solvents were purchased from VWR Chemicals in HPLC grade. Deuterated NMR solvents were purchased from Deutero. Water was purified with a Merck Millipore Mili‐Q filter system. Reversed‐phase column chromatography was performed on C18 ec silica (Macherey & Nagel, 100–50 μm). DMEM/High Glucose/ GlutaMAX™, penicillin streptomycin (P/S), 0.05 % Trypsin‐EDTA were purchased from Fisher Scientific, Fetal Bovine Serum (FBS) from PAN‐Biotech and phosphate buffered saline (PBS) from Biozym. DOTAZA has been synthesized according to a literature procedure.^[31]^

**Equipment and Purification**. NMR was performed on a Bruker Avance I 400 MHz and a Bruker Avance I 500 MHz. Chemical shifts (δ) are expressed in parts per millions (ppm). Analytical HPLC‐MS was performed on Agilent HPLC System 1260 Infinity II with a EC 150/2 Nucleodur C18 HTec, 5 μm or ZIC®‐pHILIC (Macherey & Nagel, 5 μm), linked to a Bruker ESI mass spectrometer. ICP‐MS was performed in triplicate on an Agilent Technologies 7800x series ICP‐MS (Agilent Technologies Inc., Santa Clara, USA) equipped with a quadrupole mass analyzer. Prior to measurement, the ICP‐MS setup was tuned with a mixture of Ce, Co, Li, Tl and Y at a concentration of 1 μg/L (Agilent Technologies Inc., Santa Clara, USA). External calibration was performed using mixed element standards purchased from Merck KGaAA and PerkinElmer® Inc. Calibration solutions containing Gd and Cu at concentrations of 0 to 10 ppb and 0 to 1000 ppb, respectively, were prepared freshly (parts per billion, equivalent to μg/L).

**Relaxometric studies**. The *T*_1_‐relaxation times were measured in triplicates on a Bruker microspect 60 mq relaxometer at 1.41 T and 310 K. For every compound 15 points were acquired in intervals between 5 ms and 400 ms at set Gd‐concentration (6 mm, 3.2 mm, 0.7 mm). Gadolinium molar concentration was determined based on ICP‐MS. Relaxivities were derived by linear regression from reciprocal plotting of measured *T*_1_‐times against the verified concentration c.

**Transchelation assay with 200‐fold excess of EDTA**. The complex was dissolved in Milli‐Q water (100 μL). EDTA solution (500 μL, 200‐fold excess, 0.0432 mm) in HEPES (2 mm, pH 7.4) was added. The solution was stored at room temperature. After 0 h, 2 h, 4 h, 6 h, 8 h and 36 h, 15 μL of the solution were analyzed by HPLC‐MS (ESI, positive mode). A sample of pure Gd‐DOTA‐NOx was measured as an external standard at time points 0 h and 36 h hours to calibrate the amount of intact Gd‐complex to 100 %. The amount of intact complex was determined by integration of peaks in the EIC (extracted ion chromatogram) for [M+3H]^3+^, HPLC: ZIC®‐pHILIC (Macherey & Nagel), 150×2.1 mm ID, 5 μm particles, H_2_O/CH_3_CN 98 : 2+0.05 % HCO_2_H →5:95+0.05 % HCO_2_H, 15 min, 0.2 mL/min.

**Cytotoxicity studies**. 10×10^3^ tumoral human cells (HeLa) were incubated for 24 h in a 96‐well plate at 37 °C and 5 % CO_2_ with complete DMEM media (10 % FBS and 1 % P/S). After this period, different concentration of Gd‐DOTA‐NOx dispersed in cell media were added. Three measurements were done for each concentration. Then, after another 24 h of incubation at 37 °C and 5 % CO_2_, cells were washed three times with PBS. A total of 100 μL from a 10 % solution of resazurin (7‐hydroxy‐10‐oxidophenoxazin‐10‐ium‐3‐one) in cell media was added into the wells. After 3 h of incubation, the fluorescence intensity was measured (excitation 560 nm, emission 590 nm) using a Tecan infinite M200 plate‐reader and i‐Control 1.4 software. This test is based on the irreversible reduction of resazurin to the pink and highly fluorescent resorufin. To analyze the data, the average of the background was subtracted from the maximum value. Fluorescence values obtained from control wells containing cells incubated without the addition of Gd‐DOTA‐NOx were considered as 100 % viable cells.

**Synthesis. Propargyl‐*N*‐oxide 2**. *N*,*N*‐Dimethylprop‐2‐yn‐1‐amine **1** (0.5 g, 6 mmol) was dissolved in 3 mL H_2_O_2_ (aqueous 30 %) under cooling at 0 °C. The reaction solution was stirred for 1 h at 0 °C. Excess MnO_2_ was added and the mixture was stirred for 3 h at rt. After checking the complete reduction of H_2_O_2_ using peroxide test strips, the solution was filtered. The product was further used as an aqueous solution. ESI‐HRMS (m/z): calc. for C_5_H_9_NO: 100.0757, found: 100.0756 [M+H]^+^. ^1^H NMR (600 MHz, D_2_O) δ=4.21 (d, *J*=2.5 Hz, 2H, H‐1), 3.30 (s, 6H, H‐4), 3.08 (t, *J*=2.5 Hz, 1H, H‐3). ^13^C NMR (151 MHz, D_2_O) δ=79.4 (C‐3), 72.9 (C‐2), 60.9 (C‐1), 57.1 (C‐4).

**Gd‐DOTAZA**. DOTAZA (112 mg, 0.165 mmol, 1 equiv.) was dissolved in NH_4_OAc buffer (8.0 mL, pH=5.5) and subsequently Gd_2_O_3_ (65 mg, 0.18 mmol, 1.1 equiv.) was added. The mixture was stirred at 90 °C for 3 h. The crude product was freeze‐dried and the crude product was purified by column chromatography on silica gel (RP‐C_18_ec, H_2_O/CH_3_CN with 0.1 % HCO_2_H, gradient: 98 : 0→0 : 98 v/v). The compound Gd‐DOTAZA (108.8 mg, 0.106 mmol, 79 %) was obtained as a colorless powder. ESI‐HRMS (m/z): calc. for C_24_H_36_GdN_16_O_8_: 835.2216, found: 835.2222 [M+H]^+^.

**Gd‐DOTA‐NOx**. Gd‐DOTAZA (4 mg, 4.8 μmol, 1 equiv.), CuI (0.15 mg, 0.8 μmol, 0.17 equiv.), TBTA (0.58 mg, 1 μmol, 0.21 equiv.) and NaAsc (0.6 mg, 3 μmol, 0.61 equiv.) were dissolved under N_2_‐atmosphere in degassed *t*‐BuOH/H_2_O/DMF (2 : 7 : 1, 3 mL) and propargyl‐*N*‐oxide **2** (15 μL aqueous solution) was added. The solution was stirred at 50 °C for 1 h. The solvent was removed *in vacuo* and the crude product was purified by RP‐Pad on silica gel (RP‐C_18_ec, H_2_O/CH_3_CN with 0.1 % HCO_2_H, gradient: 98 : 0→0 : 98 v/v). The compound Gd‐DOTA‐NOx (5 mg, 4.1 μmol, 91 %) was obtained as a yellow oil. ESI‐HRMS (m/z): calc. for C_44_H_72_GdN_20_O_12_: 616.2513, found: 616.2518 [M+3H]^2+^.

**Eu‐DOTAZA**. DOTAZA (100 mg, 0.147 mmol, 1 equiv.) was dissolved in NH_4_OAc buffer (5.0 mL, pH=5.5) and subsequently EuCl_3_ (42 mg, 0.162 mmol, 1.1 equiv.) was added. The mixture was stirred at 90 °C for 3 h. The crude product was freeze‐dried and the crude product was purified by column chromatography on silica gel (RP‐C_18_ec, H_2_O/CH_3_CN with 0.1 % HCO_2_H, 98 : 0→0 : 98 v/v). The compound Eu‐DOTAZA (80 mg, 0.097 mmol, 66 %) was obtained as a colorless powder. ESI‐HRMS (m/z): calc. for C_24_H_36_EuN_16_O_8_: 831.2266, found: 831.2283 [M+2H]^+^.

**Eu‐DOTA‐NOx**. Eu‐DOTAZA (15 mg, 20 μmol, 1 equiv.), CuI (0.6 mg, 3.4 μmol, 0.17 equiv.), TBTA (2.3 mg, 4 μmol, 0.21 equiv.) and NaAsc (2.4 mg, 1.2 μmol, 0.61 equiv.) were dissolved under N_2_‐atmosphere in degassed *t*‐BuOH/H_2_O/DMF (2 : 7 : 1, 3 mL) and propargyl‐*N*‐oxide **2** (70 μL aqueous solution) was added. The solution was stirred at 50 °C for 1 h. The solvent was removed *in vacuo* and the crude product was purified by RP Pad on silica gel (RP‐C_18_ec, H_2_O/CH_3_CN with 0.1 % HCO_2_H, gradient: 98 : 0→0 : 98 v/v). The compound Eu‐DOTA‐NOx (13 mg, 10.6 μmol, 53 %) was obtained as a yellow oil. ESI‐HRMS (m/z): calc. for C_44_H_72_EuN_20_O_12_: 1227.5002, found: 1227.5019 [M+2H]^+^.

**Gd‐DOTAZA‐NMe_2_**. Gd‐DOTAZA (15 mg, 20 μmol, 1 equiv.), CuI (0.3 mg, 1.6 μmol, 0.17 equiv.), TBTA (1.2 mg, 2 μmol, 0.21 equiv.) and NaAsc (1.2 mg, 6 μmol, 0.61 equiv.) were dissolved under N_2_‐atmosphere in degassed *t*‐BuOH/H_2_O/DMF (2 : 7 : 1, 3 mL) and propargylamine **1** (30 μL, 44 μmol, 4.5 equiv.) was added. The solution was stirred at 50 °C for 1 h. The solvent was removed *in vacuo* and the crude product was purified by RP Pad on silica gel (RP‐C_18_ec, H_2_O/CH_3_CN with 0.1 % HCO_2_H, gradient: 98:0 → 0 : 98 v/v). The compound Gd‐DOTAZA‐Amine (8 mg, 6.5 μmol, 68 %) was obtained as a yellow oil. ESI‐HRMS (m/z): calc. for C_44_H_72_GdN_20_O_8_: 1168.5229, found: 1168.5217 [M+2H]^+^.

## Supporting Information

The authors have cited additional references within the Supporting Information.[^30^, ^31^]

## Conflict of interests

The authors declare no conflict of interest.

1

## Supporting information

As a service to our authors and readers, this journal provides supporting information supplied by the authors. Such materials are peer reviewed and may be re‐organized for online delivery, but are not copy‐edited or typeset. Technical support issues arising from supporting information (other than missing files) should be addressed to the authors.

Supporting Information

## Data Availability

The data that support the findings of this study are available from the corresponding author upon reasonable request.
