# Supplementary material for: Decoration of 1,4,7,10‐tetraazacyclododecane‐1,4,7,10‐tetraacetic acid (DOTA) with N‐oxides increases the T 1 relaxivity of Gd‐complexes
Source: ChemistryOpen. 2024 Jan 15;13(7):e202300298. doi: 10.1002/open.202300298 (PMC11230940; doi:10.1002/open.202300298)
Supplement: Supplementary file 1 — Supporting Information [file OPEN-13-e202300298-s001.pdf]

ChemistryOpen

Supporting Information

Decoration of 1,4,7,10-tetraazacyclododecane-1,4,7,10-tetraacetic acid (DOTA) with *N*-oxides increases the T_1 relaxivity of Gd-complexes

Svenja Kerpa, Verena R. Schulze, Malte Holzapfel, Lina Cvancar, Markus Fischer, and Wolfgang Maison*

Supporting Information

Decoration of 1,4,7,10-tetraazacyclododecane-1,4,7,10-tetraacetic acid (DOTA) with *N*-oxides increases the T_1 relaxivity of Gd-complexes

Svenja Kerpa, Verena R. Schulze, Malte Holzapfel, Lina Cvancar, Markus Fischer and Wolfgang Maison*

Contents

Table S1. Cytotoxicity test. 2

Analytical data 3

Propargyl-*N*-oxide **2** 3

Gd-DOTAZA 4

Gd-DOTAZA-NOx 5

Eu-DOTAZA 6

Eu-DOTA-NOx 7

Gd-DOTAZA-NMe_2 9

Table S1. Cytotoxicity test. Results of the cytotoxicity evaluation of Gd-DOTA-NOx with HeLa cells. Cell viability was measured by colorimetric measurement of resazurin reduction as described in the experimental. The standard deviation (SD) is given for triplicate measurements.

Gd-DOTA-NOx concentration (μM)	Cell viability (%)	SD (%)
0,488	98	0,5
0,977	102	4,4
1,953	103	4,2
3,906	103	3,3
7,813	103	3,4
15,63	103	2,4
31,25	99	3,3
62,50	94	0,9
125,0	88	2,5
250,0	82	6,1
500,0	73	7,6
1000	40	14,4

Analytical data

Propargyl-*N*-oxide **2**

^{1}H-NMR spectrum of propargyl-*N*-oxide **2**

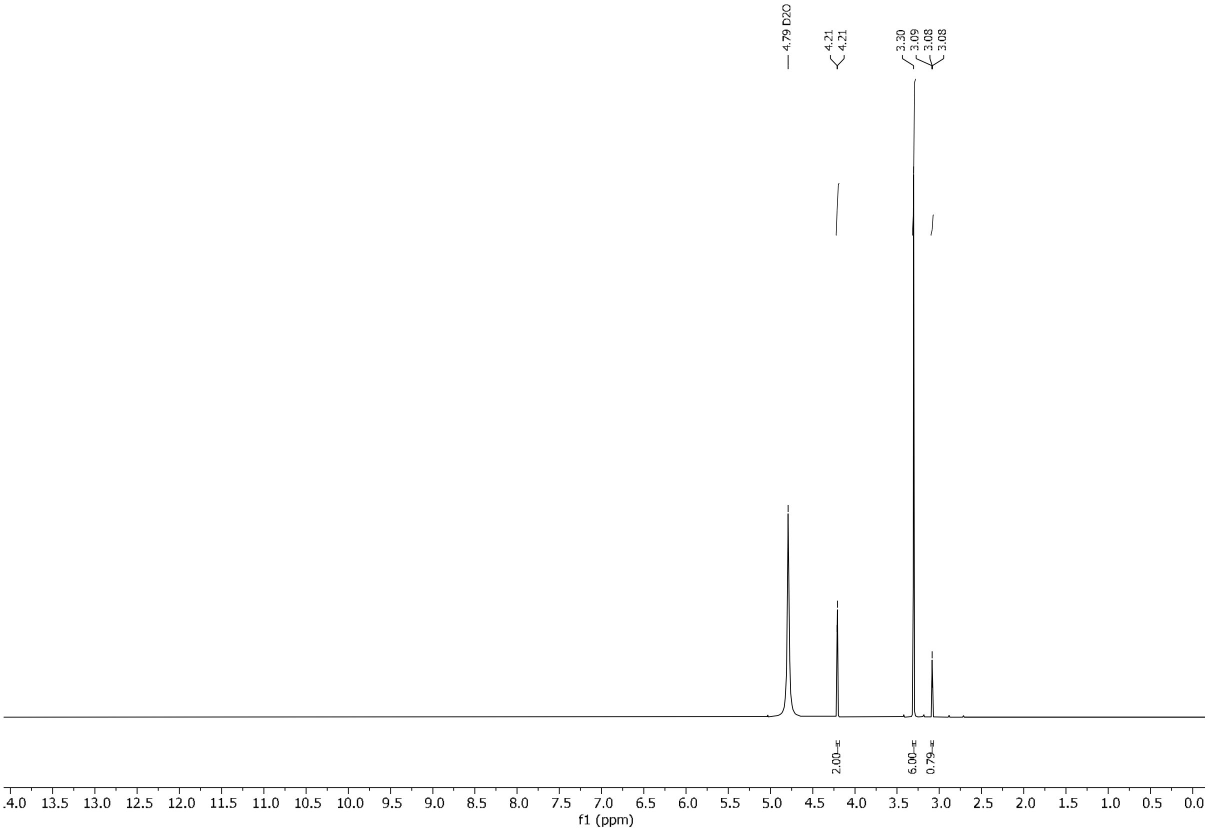

^{13}C-NMR spectrum of propargyl-*N*-oxide **2**

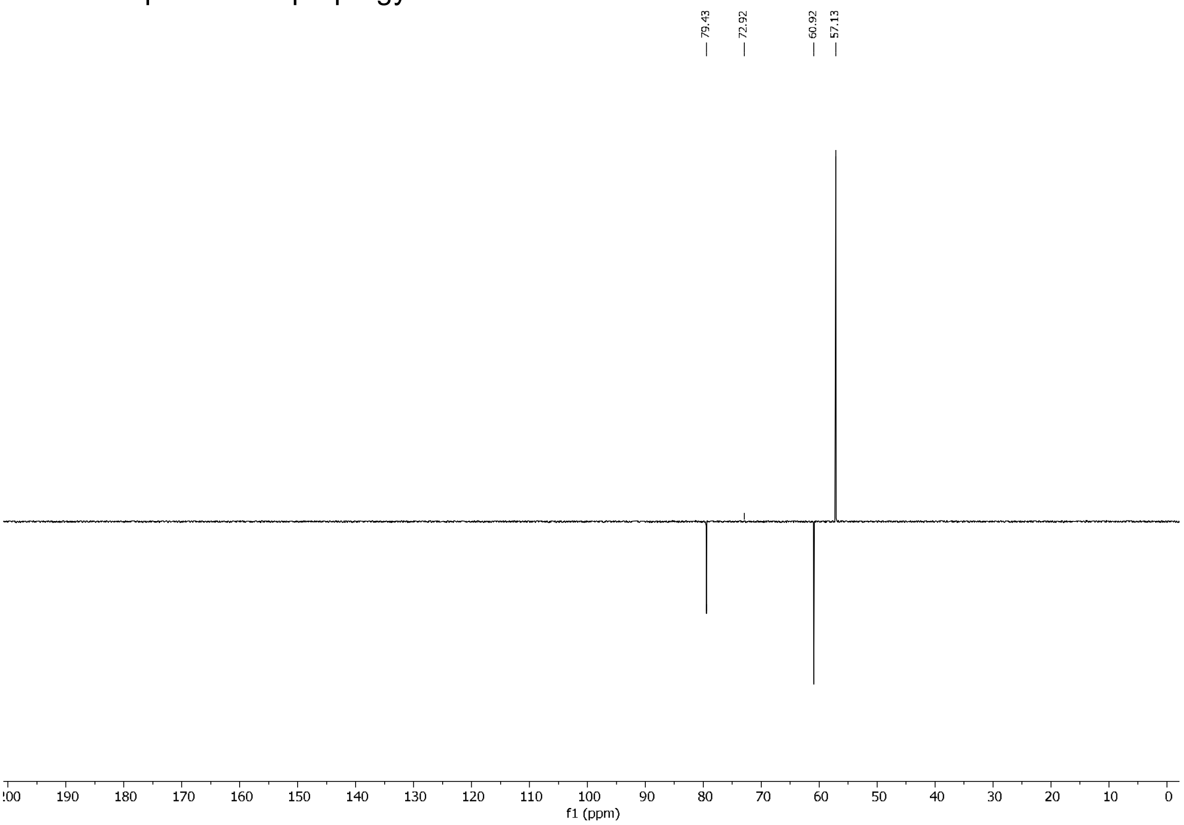

Gd-DOTAZA

LC/MS of Gd-DOTAZA

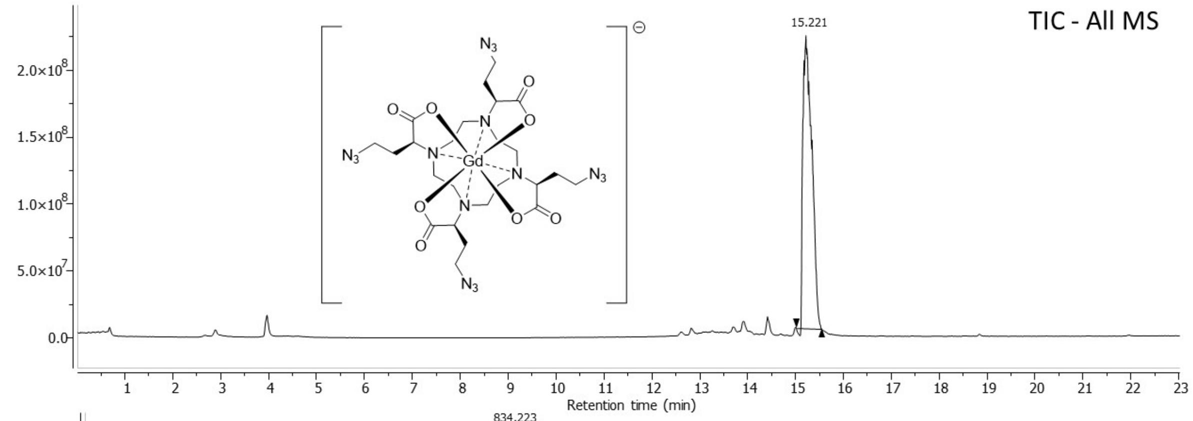

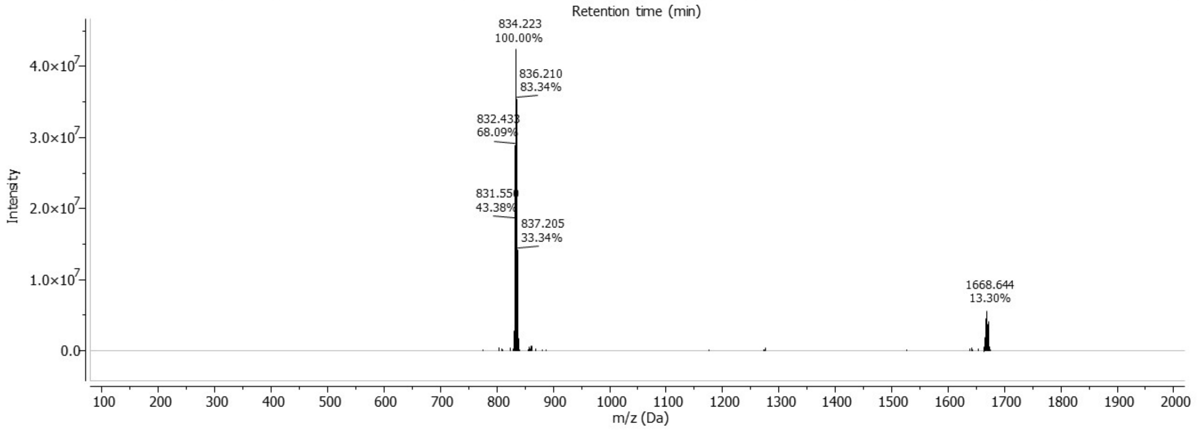

HPLC conditions: A Poroshell 120 C18 RP column from Agilent (4.6 × 100 mm) was used as stationary phase and the following gradient of MeCN in H_2O with 0.1 % formic acid was used as mobile phase:

Time	H_2O	MeCN	Flow
3	95	5	0.25
20	5	95	0.25
21	95	5	0.25
24	95	5	0.25

Gd-DOTAZA-NOx

LC/MS of Gd-DOTA-NOx

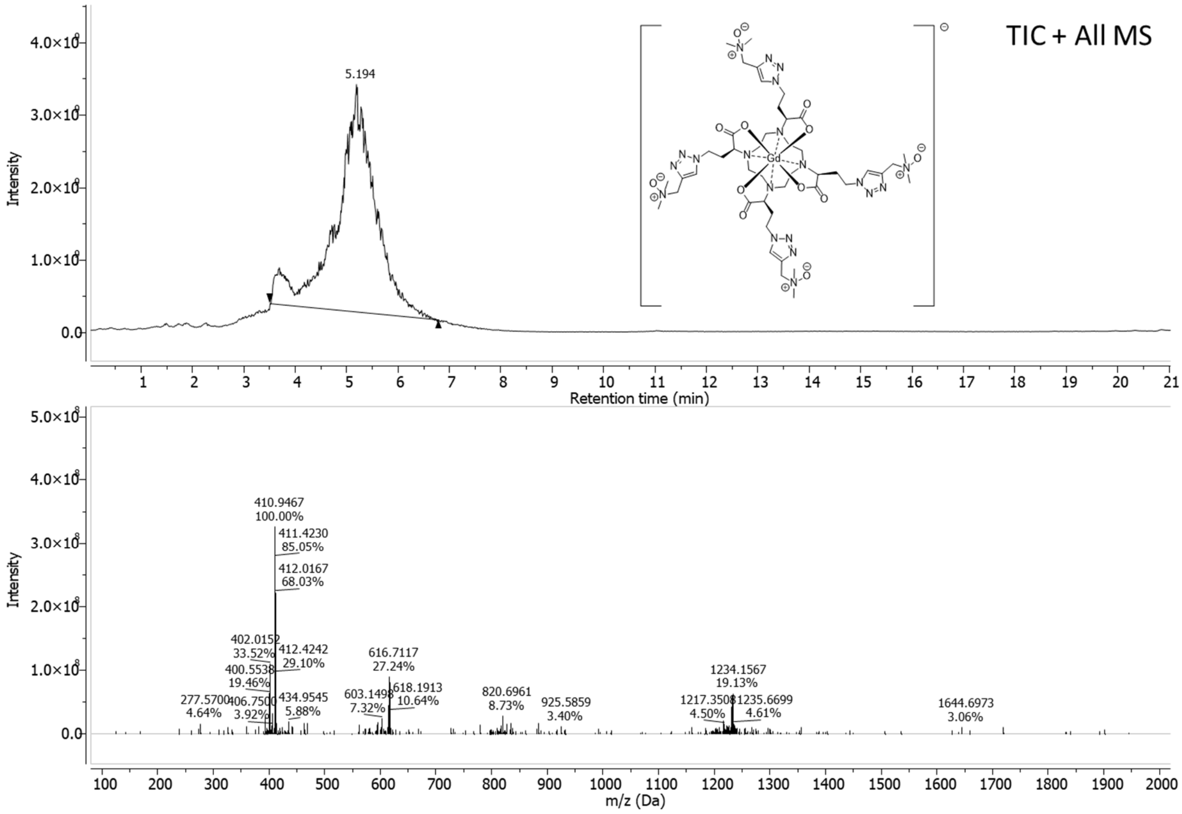

HPLC conditions: A Poroshell 120 C18 RP column from Agilent (4.6 × 100 mm) was used as stationary phase and the following gradient of MeCN in H_2O with 0.1 % formic acid was used as mobile phase:

Time	Water	Acetonitrile	Flow
2	95	5	0.25
17	5	95	0.25
19	95	5	0.25
21	95	5	0.25

Eu-DOTAZA

LC/MS of Eu-DOTAZA

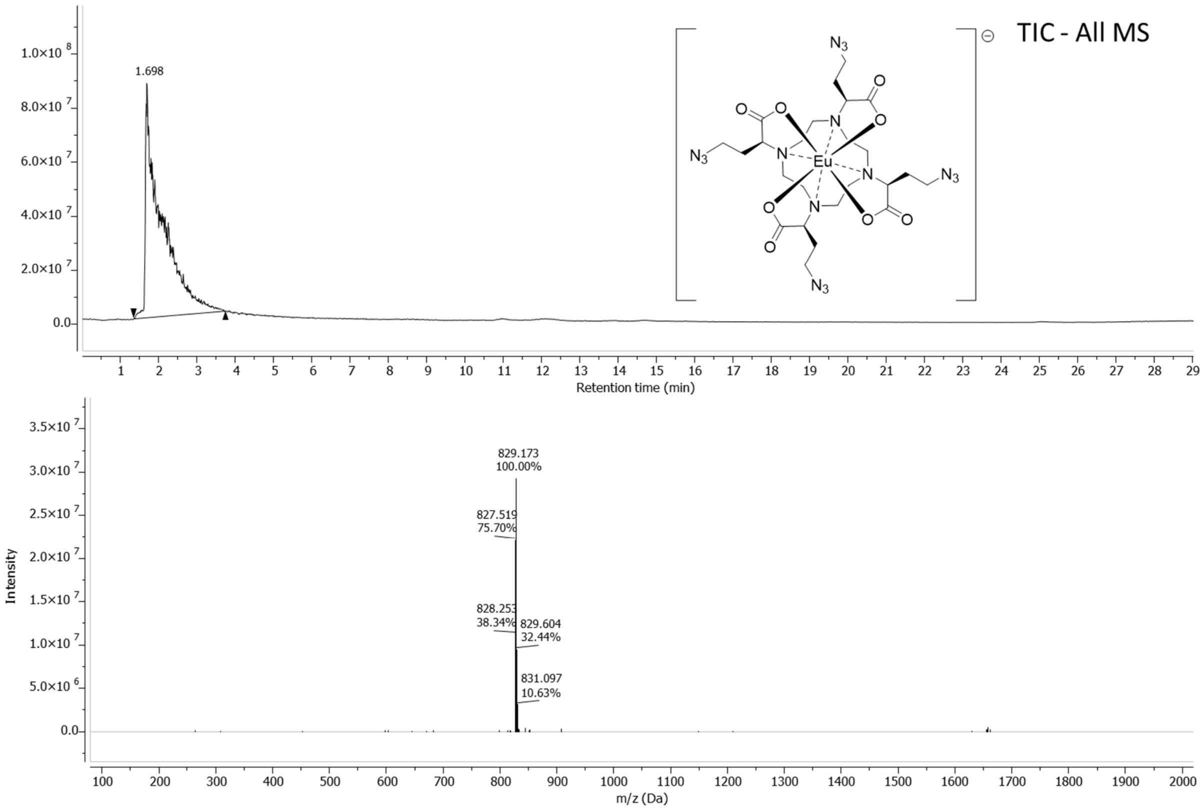

HPLC conditions: ZIC-pHILIC column from Macherey-Nagel (2.1 × 150 mm) was used as stationary phase and the following gradient of MeCN in H_2O with 0.1 % formic acid was used as mobile phase:

Time	Water	Acetonitrile	Flow
3	15	85	0.25
18	45	55	0.25
22	45	55	0.25
23	15	85	0.25
29	15	85	0.25

Eu-DOTA-NOx

LC/MS of Eu-DOTA-NOx

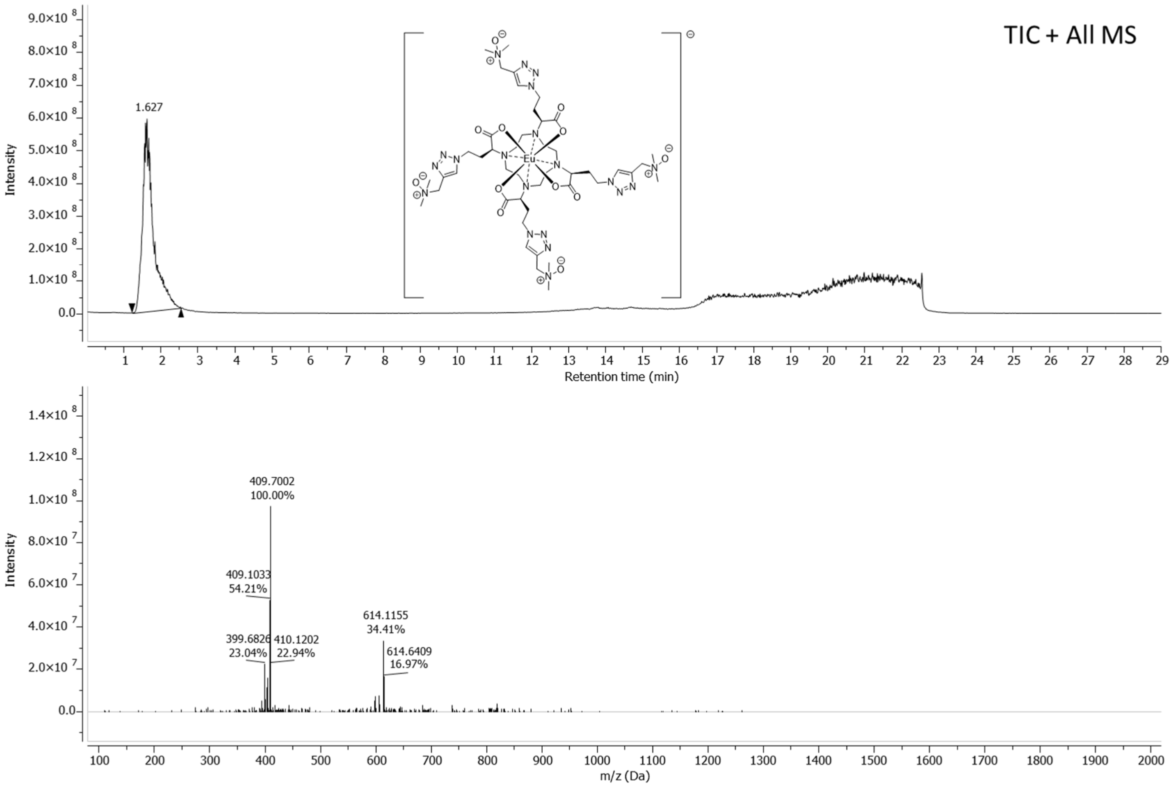

HPLC conditions: A Poroshell 120 C18 RP column from Agilent (4.6 × 100 mm) was used as stationary phase and the following gradient of MeCN in H_2O with 0.1 % formic acid was used as mobile phase:

Time	Water	Acetonitrile	Flow
3	95	5	0.25
18	5	95	0.25
22	5	95	0.25
23	95	5	0.25
29	95	5	0.25

^{1}H-NMR spectrum of **Eu-DOTA-NOx**

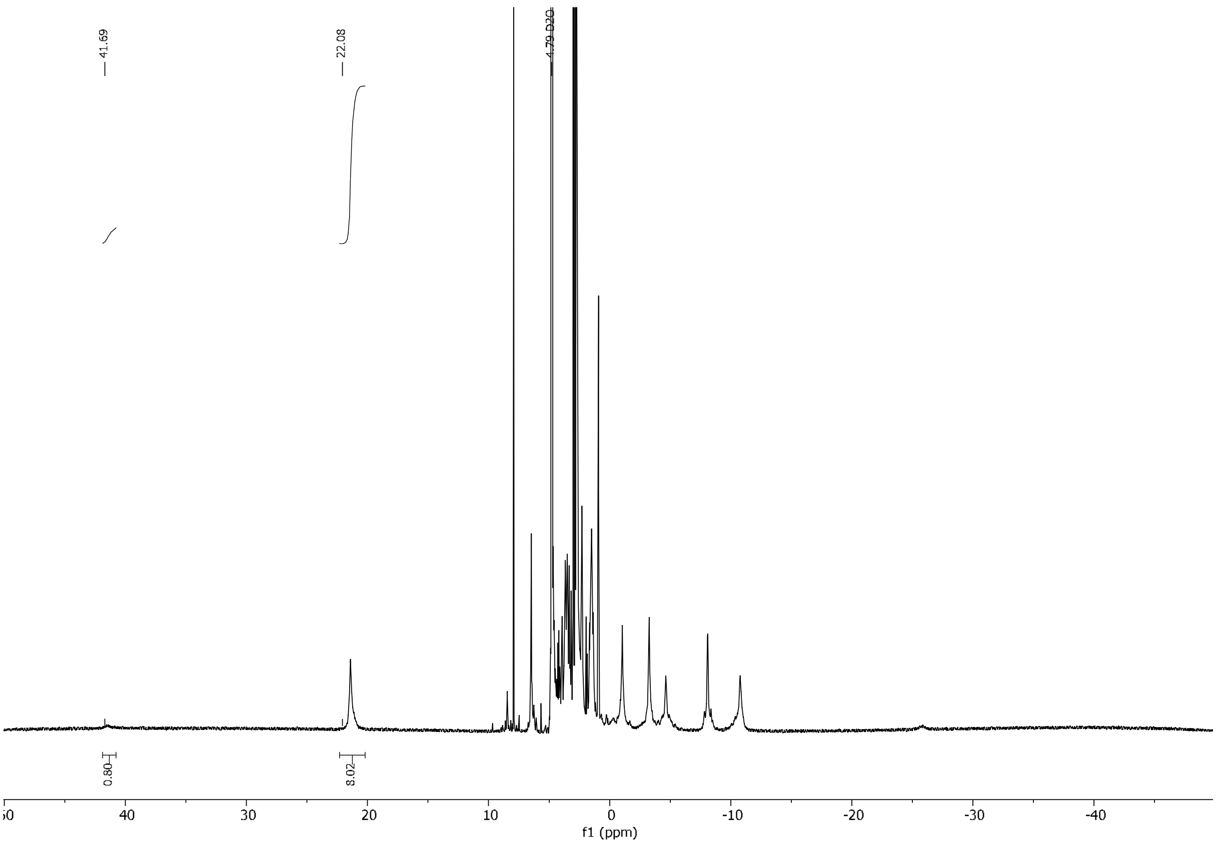

Gd-DOTAZA-NMe$_2$

LC/MS of Gd-DOTAZA-NMe2

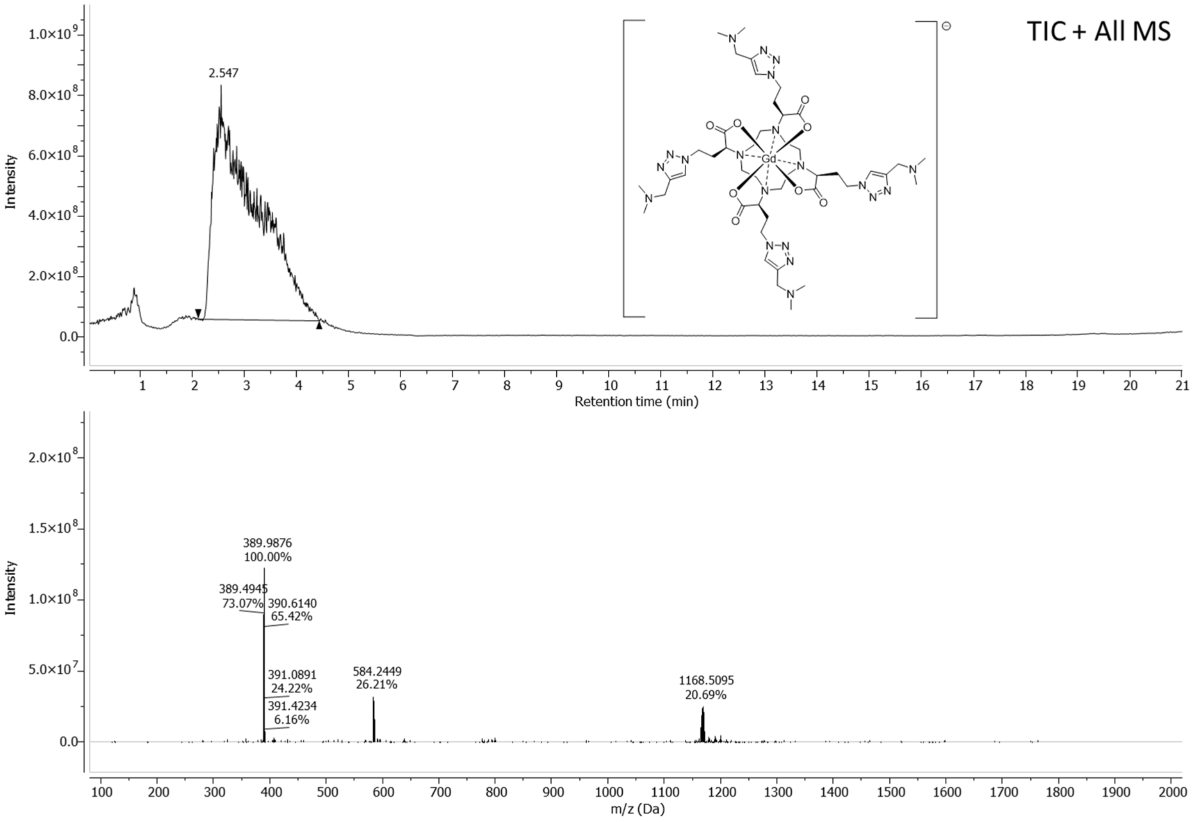

HPLC conditions: A Poroshell 120 C18 RP column from Agilent (4.6 × 100 mm) was used as stationary phase and the following gradient of MeCN in H_2O with 0.1 % formic acid was used as mobile phase:

Time	H_2O	MeCN	Flow
3	95	5	0.25
20	5	95	0.25
21	95	5	0.25
24	95	5	0.25
